# A High‐Performance Application Specific Integrated Circuit for Electrical and Neurochemical Traumatic Brain Injury Monitoring

**DOI:** 10.1002/cphc.201701119

**Published:** 2018-03-09

**Authors:** Ilias Pagkalos, Michelle L. Rogers, Martyn G. Boutelle, Emmanuel M. Drakakis

**Affiliations:** ^1^ Department of Bioengineering Imperial College London UK

## Abstract

This paper presents the first application specific integrated chip (ASIC) for the monitoring of patients who have suffered a Traumatic Brain Injury (TBI). By monitoring the neurophysiological (ECoG) and neurochemical (glucose, lactate and potassium) signals of the injured human brain tissue, it is possible to detect spreading depolarisations, which have been shown to be associated with poor TBI patient outcome. This paper describes the testing of a new 7.5 mm^2^ ASIC fabricated in the commercially available AMS 0.35 μm CMOS technology. The ASIC has been designed to meet the demands of processing the injured brain tissue's ECoG signals, recorded by means of depth or brain surface electrodes, and neurochemical signals, recorded using microdialysis coupled to microfluidics‐based electrochemical biosensors. The potentiostats use switchedcapacitor charge integration to record currents with 100 fA resolution, and allow automatic gain changing to track the falling sensitivity of a biosensor. This work supports the idea of a “behind the ear” wireless microplatform modality, which could enable the monitoring of currently non‐monitored mobile TBI patients for the onset of secondary brain injury.

## 1 Introduction

Traumatic Brain Injury (TBI) can be defined as a brain trauma due to an external mechanical force.^1^ TBI is a major cause of death and disability in all age groups and the leading cause of death and disability in working people and among young adults.^2^ In the UK an estimated 238,000 people suffer a TBI each year.^3^ Most are stabilised in an intensive care unit (ICU) for extended periods before discharge, often with life‐changing disability. A third of all injury‐incurred deaths in the US relates to TBI.^4^ In the US for the period between 2002 and 2006, 1,7 million TBI incidents were reported every year. TBI was recently recognized as a “silent epidemic” worldwide by the “leading edge” Lancet Neurology Editorial,^5^, ^6^ In that same article it was highlighted that “road traffic accidents, which are already a leading cause of TBI in many parts of the world, are expected to become the third largest cause of global disease burden by 2020”. The article concludes that without advances in TBI research, “TBI is likely to remain a silent epidemic”.

The diversified pathology of TBI often evolves in the early days following the injury. The damage occurring immediately at the moment of impact is called primary brain injury. “Secondary” injury, is delayed and occurs in 30 %–40 % of TBI patients each year, whilst they are in the ICU. Secondary injuries reduce survival rate after TBI,^7^, ^8^, ^9^ and usually occur within 10–14 days post primary injury. However, as prediction of secondary brain injury onset is not currently possible, current “wait and see” management strategies are limited to attempting to optimize the physiological environment of the injured tissue by maintaining adequate perfusion pressure and reducing the energy demands of the brain by sedation.

A better approach to treat a patient suffering a TBI, is on‐line measurement of intracranial parameters to enable detection of harmful events prior to the development of irreversible tissue damage. The collection of such dynamic pathophysiological data allows for patient‐specific, dynamic treatments to be offered. There are various methods and devices commonly used in TBI monitoring and a review study by Strong et al.^7^ highlights the advantages and disadvantages of each method.

### 1.1 Spreading Depolarisation

SD waves were first identified by Leao in 1944^10^ and since then on‐line monitoring has allowed researchers to identify SD as an important dynamic secondary insult.^11^ SD waves spread out from the initial site of injury into the surrounding brain tissue “at risk” of secondary injury often cycling around the primary injury site^12^ to give a striking periodicity. SD waves are mass depolarisations of all neurons and astrocytes that propagate through the injured brain at 1–5 mm min^−1^ and are associated with poor outcome in brain trauma.^13^ The signature signal of an SD wave, as it passes a measurement point, is a slow potential change accompanied by a transient suppression of higher frequency activity that can be detected using electrical contacts placed in the brain. The duration of the suppression and the periodicity of its re‐occurrence are thought to be indicators of tissue health.^14^ SD waves have been linked to TBI^15^ and optimal ways to capture such behaviour of the injured brain tissue are discussed in the following sections.

### 1.2 Neurochemical Monitoring

The recent consensus statement from the 2014 International Microdialysis Forum^16^ states clearly that it is the assessment of dialysate glucose levels and the lactate levels that are the most clinically useful signals to guide treatment of TBI.

The method that is mostly used to measure these neurochemical signals is microdialysis. The main reason is that microdialysis enables the measurement, without sensors being implanted into the injured tissue. Classic microdialysis is a sampling technique with sampling rate of 1 hour. However, for applications such as TBI, in which chemical changes occur in shorter time frame than an 1 hour, the information of interest is lost. Rapid‐sampling microdialysis (rsMD) is an on‐line flow injection technique with samples taken each minute. It enabled the measurement of glucose and lactate leading to the identification of depolarisation‐like events.^17^ Recently, we have combined amperometric biosensors, ion selective electrodes and microfluidic chips to allow multiple analytes to be measured continuously, in real‐time directly in the dialysis stream (continuous microdialysis, coMD).^18^, ^19^

**Figure 1:**
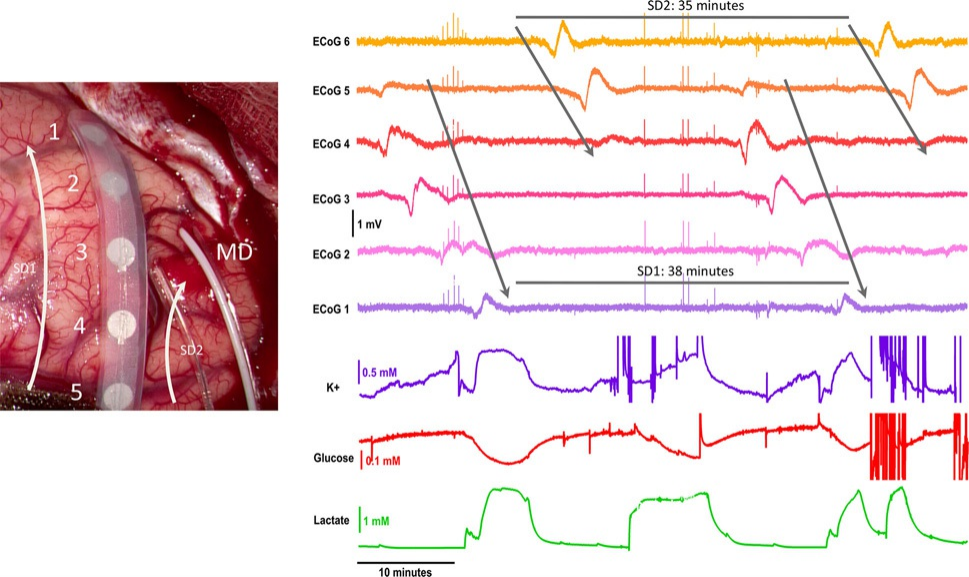
Left: A) Picture of the injured brain tissue with six‐contact ECoG strip electrode and microdialysis (MD) probe on top. Right: B) Illustration of the identified SD waves through concurrent recordings of ECoG (electrical) and glucose/lactate/potassium (neurochemical) signals, as presented in Ref. [19]. Image copyright Boutelle Group.

Basal dialysate levels of glucose and lactate are typically between 0.4 and 0.5 mm and 0.5 and 2 mm respectively.^20^ During an SD event, the level of glucose becomes depressed and in a damaged tissue the local demand for glucose and oxygen often outstrips supply and the level of glucose becomes depressed (from 93 to 18 μm) and the level of lactate transiently increases (from 5 to 100 μm) as a huge metabolic demand is placed upon the tissue to re‐polarise cell membranes.^20^ A neurochemical signature has been recently defined using coMD^18^ (see Figure 1). Local levels of glucose and lactate are key to interpreting the health state of the tissue. If the SD event repeats, typically SDs occurring within clusters, the tissue may not be able to fully recover between SD events and therefore the size of the damaged area of the brain tissue expands, leading to a poorer patient outcome.

### 1.3 ECoG Monitoring

SD waves have been detected with electrocorticography (ECoG) where electrodes are placed under the dura directly on the exposed cortical surface.

There are two types of ECoG (a) the strip electrode^21^ and (b) the depth electrode.^15^ The strip electrode can only be used following craniotomy surgery, and hence is restricted to severely injured patients. On the other hand, the depth electrode is placed to the cortex through a burr hole. Burr hole surgery is a less invasive surgery than craniotomy, can be performed on the intensive care ward, and hence could be used with more mobile patients if suitable instrumentation was available. There are now preliminary report of devices which combine neurochemical measurement and electrophysiology.^22^

With the use of either type of electrode, the SD wave is seen as a large negative slow potential change, followed by a long period of attenuated electrical activity (see Figure 1). Such brain activity is captured within two bands of interest. The low band frequency close to DC, which is also referred to as quasi‐DC, and the high frequency band with range of frequencies 0.5 Hz to 30 Hz.^23^ It has been experimentally studied that for long periods of monitoring, in order to avoid the DC drift of the signal, the ECoG signal is AC coupled with a cut‐off frequency of 0.02 Hz.^15^ Notably, the low band frequency gets values close to DC and not actual DC. The utility of wireless electronics for patient neurochemical monitoring with electrochemistry is recognised, but to date has been little explored. The groups of Garris and Lee produced the surface mount Wireless Instantaneous Neurotransmitter Concentration Sensing (WINCS) device^24^, ^25^ capable of carrying out fast cyclic voltammetry (FSCV) in both rats and patients. The challenge here was for fast data throughput given the typical 10 ms of a FSCV scan. Recently, the WINCS device has been improved to make WINCS Harmoni^26^ designed to link FSCV with deep brain stimulation. The system includes a monolithic 4 channel simultaneous delta‐sigma analogue to digital converter that delivers 12.8 MByte per second transfer rate of 16 bit data, equivalent to 100 Ksamples/ second.^27^ The authors have used this to monitor adenosine in the human brain following electrical stimulation. The WINCS Harmoni device is optimised for speed, as required for FSCV. We present here a novel, entirely monolithic device optimised for low voltage and current performance, as required by neurophysiological recording, ion selective electrodes and amperometric biosensors.

## 2 Results

The overall testing strategy of the fabricated ASIC aims at assessing the performance of every presented part. In particular we present test voltage measurements for ECoG and test current measurements for amperometry. Test voltage measurements put emphasis on highlighting the robustness of the fabricated ASIC against process parameter variations and its ability to interface with test ECoG data of varying amplitude. Test current measurements put emphasis on highlighting the ability of our amperometric channels to offer electronically controlled, switched‐capacitor‐based (not ohmic resistor‐based), low‐noise input current to output voltage conversion; the transimpedance gain values of our amperometric channels are set to 1, 10 and 100 mV pA^−1^. This is achieved without resorting to noisy, large valued ohmic resistors thanks to the adoption of the switched‐capacitor approach (see also Experimental Methods section). Various methods and techniques have been adopted throughout the testing period of the proposed TBI ASIC. The results confirm the high performance of the ASIC and its suitability for the collection of neurochemical TBI physiological signals. The successful operation of our TBI chip supports the idea of a wireless microplatform placed “behind‐the‐ear” and enabling the monitoring of currently non‐monitored mobile TBI patients for the onset of secondary brain injury.

### 2.1 Test Voltage Measurements for ECoG

**Figure 2:**
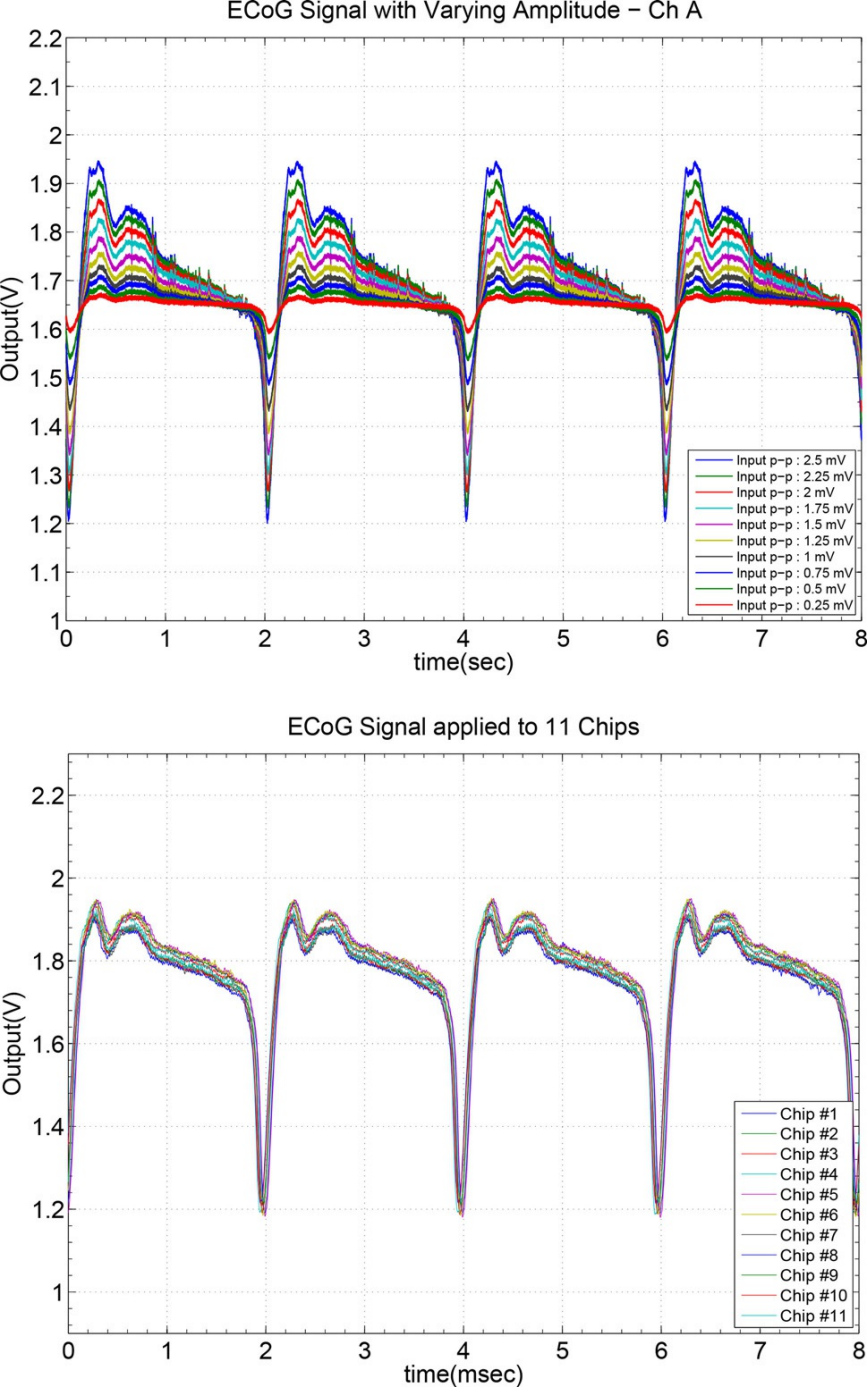
Response of the ECoG Channel A (low pass filtered at 350 Hz) for a played back ECoG signal with a peak to peak value ranging from 250 μV to 2.5 mV with a step of 250 μV. Bottom graph shows the measurement of an 2.5 mV peak to peak ECoG artificial signal across 11 fabricated microchips (variability results). Observe the very low spread of the response across 11 fabricated chips which verifies the robustness of our ASIC thanks to its high‐quality design and physical layout.

The performance of the voltage Analog Front End (AFE) was evaluated by means of the measurement of an anonymised played back ECoG signal collected in the ICU and provided by our medical collaborators as also explained in.^28^ The test waveform was applied at the input of the ECoG voltage channels of our ASIC by means of the Agilent 3320 function generator which was loaded with the anonymised ECoG data and was programmed to play them back keeping control of the amplitude and the frequency of the waveform. The importance of this experiment was to highlight the capability of the ECoG voltage AFE channels to record the dynamics of an ECoG signal due to an SD wave. The first two graphs of Figure 2 illustrate the capturing of ECoG signals with variable amplitude from the two channels with different bands of interest. As can be seen the low band of interest (ECoG channel B) captures the slow dynamics while neglecting the brain activity in higher frequencies, while the high band of interest channel (ECoG channel A) having a low pass filter (LPF) cut‐off frequency at 500 Hz incorporates information from the brain activity in these higher frequencies.

In Figure 2 a 0.5 Hz ECoG input has its peak‐to‐peak value varying from 250 μV to 2.5 mV with a step of 250 μV and the outputs of the ECoG channels A and B were recorded. Channel B, which is optimised to capture ECoG characteristics in lower frequencies, recorded the slow dynamics of the test signal disregarding any high frequency components present at the input signal. On the other hand, channel A, which is optimised to capture brain activity present in higher frequencies, captured both the slow and the high frequency characteristics of the input signal thus resulting into a more “spiky” behaviour.

Fabricated microelectronic circuits suffer from “mismatch errors” due to process parameter variations. Minimisation of mismatch errors is possible by means of extremely high‐quality and symmetry physical layout of the integrated circuits (eg. adoption of common‐centroid layout techniques) and manifests itself by small only variations in the response of many copies of dies of the same design when each one of the dies/designs is excited by the same input. A small spread in such inter‐chip variability results verifies the robustness of the tested design/ASIC. Thus, in order to prove the robustness and the consistency of the proposed fabricated microchip, the ECoG channel A performance of multiple microchips was evaluated. A chosen ECoG signal scenario with a frequency of 500 Hz and medium value of input peak‐to‐peak value of 2.5 mV, was set at the input of eleven microchips and the performance is presented in Figure 2. The identical output behaviour across eleven microchips verifies the enviable robustness of the fabricated microelectronic system.

### 2.2 Test Current Measurements for Amperometry

**Figure 3:**
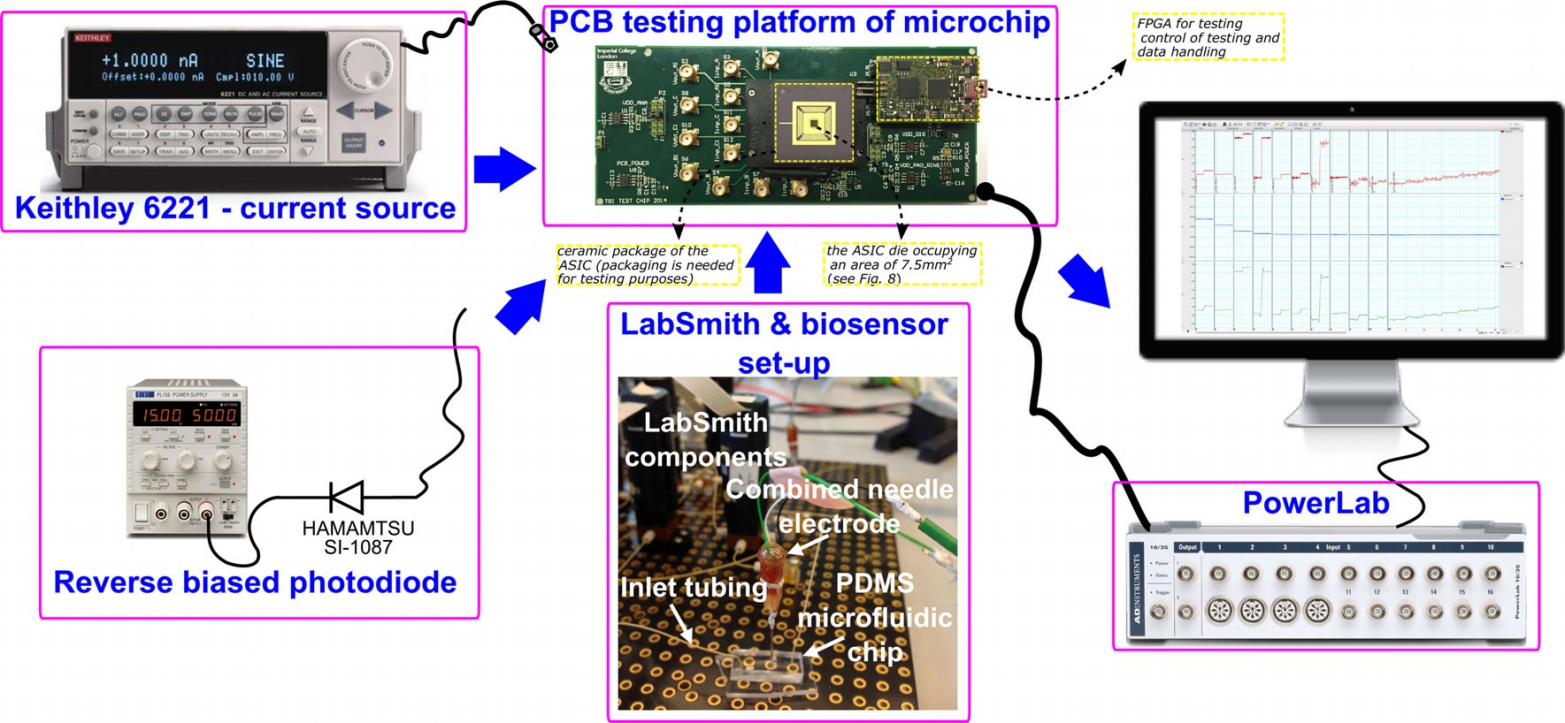
Illustration of the amperometric tests set‐up, illustrating the three types of test input sources: 1) the Keithley 6221, 2) the dark current of Hamamatsu SI‐1087 and 3) the biosensor. Labsmith is a setup of pumps that can be programmed to control the concentration of the chemical on the microfluiding chip where the biosensor is placed and measures. Powerlab is a data acquisition device by ADInstruments utilised to display the measured signals. The testing PCB (green material/photo on top of the Figure) contains the die of the ASIC (black square dot in the middle of the yellow square), which is packaged (black surrounding the yellow square) to enable and facilitate the testing of the ASIC. Several input/output testing points are shown along with a small size Field Programmable Gate Array (FPGA), which is used for testing and control of the testing purposes(it can be programmed to generate appropriate control signals and to handle the traffic of data).

The performance of the proposed circuit was evaluated in multiple ways using different input sources. As can be seen in the test set‐up block diagram of Figure 3, the electronic testing of the circuit was initially performed with the use of the Keithley 6221 as input current source. However, for exploiting the potential of the proposed circuit in the range of femto Ampere (fA), the non‐idealities of the Keithley 6221 become dominant and the measurements innacurate. Thus, an additional alternative input source, capable to reliably provide current in fA range, was used. As published in the manufacturer data sheet,^29^ the dark current of the reverse biased Hamamatsu SI‐1087 photodiode is reliably reaching down to the fA range and it was used to evaluate the amperometry performance of the ASIC in these low current ranges. Finally, a lactate biosensor was used as an input source in order to highlight the performance of the circuit under realistic scenarios and its capability to interface with real electrochemical biosensors and not just with ideal current sources.

#### 2.2.1 Test Current Input Source—Keithley 6221

**Figure 4:**
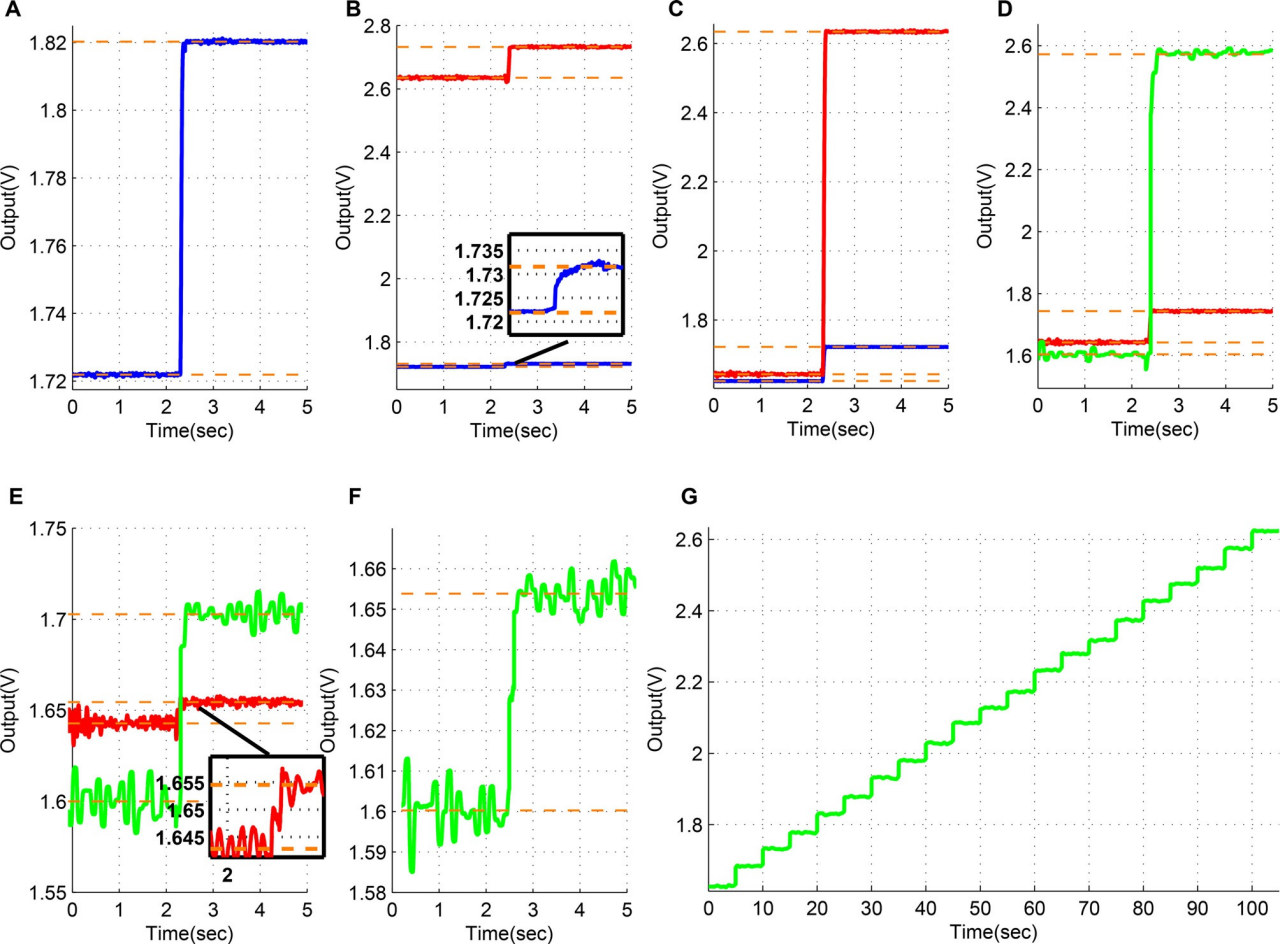
Amperometry test results for different input current steps and baselines; the blue lines correspond to a transimpedance gain of 1 mV pA^−1^, the red lines to a gain of 10 mV pA^−1^ and the green lines to a gain of 100 mV pA^−1^. Parts B, C, D and E illustrate on the same graph the response of the ASIC for two different colour‐coded transimpedance gain values. All currents are supplied by the Keithley 6221. Part A: baseline of 100 pA and a step change of 100 pA. Response for transimpedance gain value set equal to 1 mV pA^−1^ (blue). Part B: baseline of 100 pA and a step change of 10 pA. Responses for transimpedance gain values set equal to 1 (blue/inset box) and 10 (red) mV pA^−1^. Part C: baseline of 0 pA and a step change of 100 pA. Responses for transimpedance gain values set equal to 1 (blue) and 10 (red) mV pA^−1^. Part D: baseline of 0 pA and a step change of 10 pA. Responses for transimpedance gain values set equal to 10 (red) and 100 (green) mV pA^−1^. Part E: baseline of 0 pA and a step change of 1 pA. Responses for transimpedance gain values set equal to 10 (red/inset box) and 100 (green) mV pA^−1^. Part F: baseline of 0 pA and a step change of 500 fA. Response for transimpedance gain value set equal to 100 mV pA^−1^ (green). Part G: DC sweep from 0 pA to 10 pA with 500 fA step changes. Response for transimpedance gain value set equal to 100 mV pA^−1^ (green).

The Keithley 6221 is a high precision current source providing DC and AC currents in the region of 100 fA to 100 mA and in the lower range of operation and frequencies ranging from 0.1–10 Hz, the RMS noise is 80 fA.^30^ Considering that the chemical signals of interest for TBI monitoring are changing slowly, step responses with a frequency of 0.2 Hz were used to assess the ASIC. The step responses shown in Figure 4 illustrate the voltage at the output of the amperometric channels of the ASIC for a combination of input steps and baselines. Our switched‐capacitor‐based circuits converts an input current to an output voltage without employing noisy prone ohmic resistors. The equivalent transimpedance value used for this conversion can be set/controlled electronically thanks to the adoption of our switched‐capacitor approach (see also Experimental Methods section). The proposed ASIC was designed to realise transimpedance gain values of 1, 10 and 100 GΩ. In general, smaller input current values call for higher transimpedance gain values in order for the (higher) resulting output voltage to be more reliably detected and processed further down the data acquisition path.

The results shown in Figure 4 highlight the advantage of using a higher gain for lower input current levels. A current of 100 pA, 10 pA and 1 pA through 1 mV pA^−1^, 10 mV pA^−1^ and 100 mV pA^−1^ gain values, respectively each give 100 mV. For example in Figure 4 B, a 10 pA difference is detectable for a gain of 1 mV pA^−1^ as a voltage difference of 10 mV at the output, however for a gain of 10 mV pA^−1^ the resolution is clearly better as a difference of 100 mV and this gain setting for such input differences is preferred. In order to exploit the higher gain option, the level of the measured signal should be within the input ranges of every gain stage. In Figure 4 F and G it is indicated that the minimum detectable input illustrated was at 500fA. It should, however, be noted that this limitation arises from the Keithley 6221 and not from the circuit. It has been measured that the Keithley 6221 introduces a DC and AC offset in the range of 1 pA and 300 fA, respectively. It was therefore decided that different input source needs to be applied in order to evaluate any further current readout capabilities of the proposed circuit.

#### 2.2.2 Test Current Input Source—Hamamatsu SI‐1087

In order to further explore the performance of the proposed circuit for ultra low input current levels, the dark current of a photodiode was used as an input source. Dark current is defined by the leakage current that flows through the reversed biased photodiode when the photodiode operates in photoconductive mode.^31^ In photoconductive mode the current is directly proportional to the optical power received on the photodetector plus the dark current. Eliminating the optical power by performing the experiment in dark conditions, only the dark current passes through the photodiode. The dark current varies with temperature and with the bias voltage applied. Increasing the reverse bias voltage, the width of the junction increases and subsequently the dark current increases as well.

**Figure 5:**
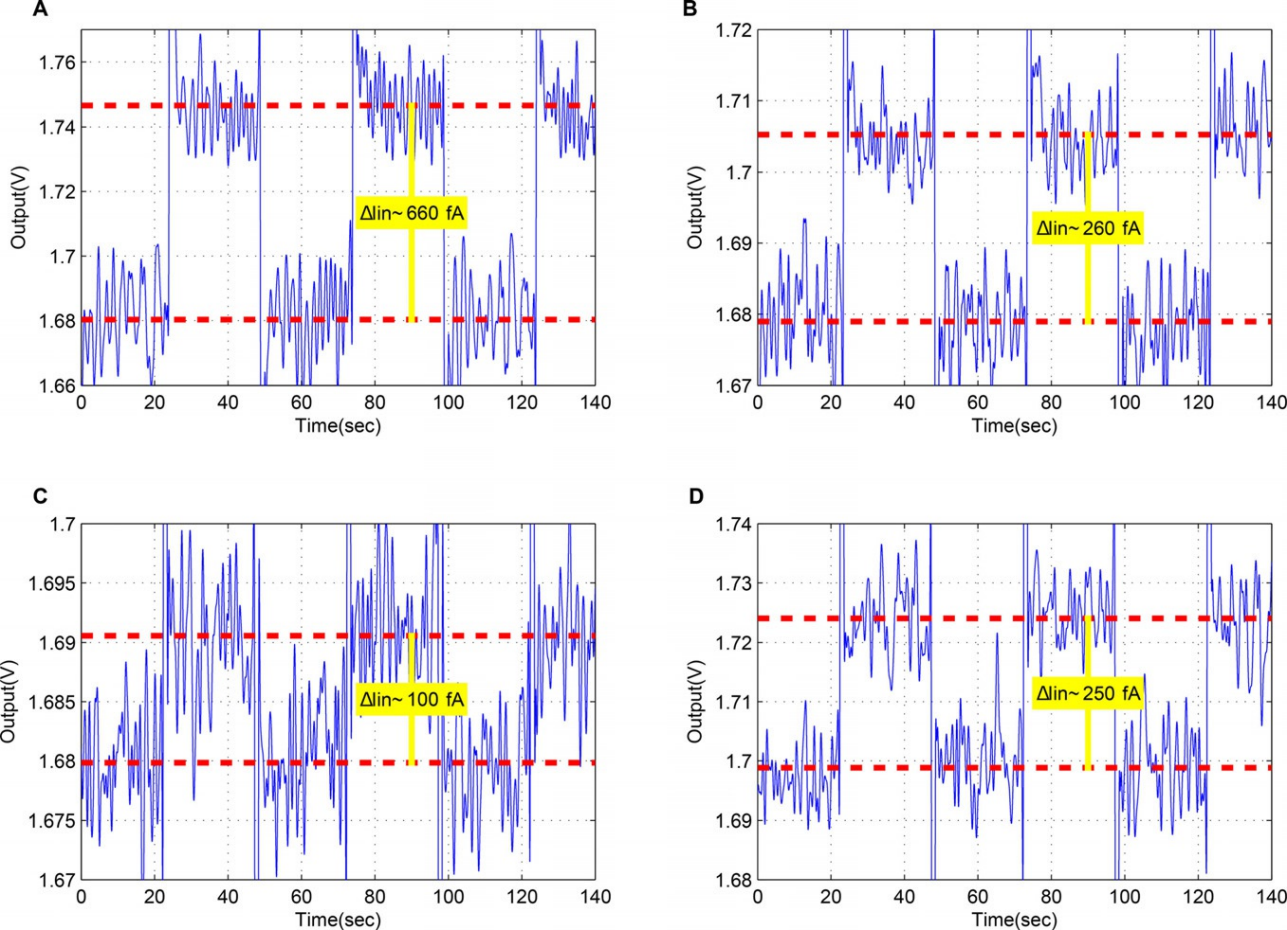
Measured test results for low frequency current pulses generated by reverse biasing of the SI‐1087 Hamamatsu photodiode. A, B and C present the response of the circuit under three different sets of reverse biasing of a single photodiode while D shows the response under the same set of basing as in C but for a 2 diodes in parallel. Part A: Dark current for reverse voltage of 50 mV and 3 V (biasing points α and δ in supporting Figure 1). Part B: Dark current for reverse voltage of 50 mV and 1 V (biasing points α and γ in supporting Figure 1). Part C: Dark current for reverse voltage of 50 mV and 300 mV (biasing points α and β in supporting Figure 1). Part D: Dark current for reverse voltage of 50 mV and 300 mV (biasing points α and β in supporting Figure 1) for two diodes.

At first, to perform the experiment efficiently, the photodiode was covered with a black tape to prevent any exposure of the photodetector in light. Then the taped photodiode was inserted in the faraday cage and it was securely hidden from the light environment. Finally, for additional reassurance the experiment took place in a dark lab, thus eliminating any potential light sources. The aim of this experiment was to showcase the potential of the proposed circuit to read current differences of 100 fA when the transimpedance gain is set to 100 mV pA^−1^. As shown in Figure 5, multiple reverse biasing sets were applied to the photodiode and the corresponding responses at the output were measured. Figure 5 A, B and C present results from the biasing of a single photodiode, while in graph D the same biasing set as in graph C is applied across two photodiodes connected in parallel confirming the output's doubling. More specifically, in Figure 5. A two values of reverse voltage where chosen, one of 50 mV and another of 3 V resulting in a dark current of ≈100 fA and ≈760 fA, respectively. In Figure 5 B the values of the reverse voltage were 50 mV and 1 V resulting to dark currents of ≈100 fA and ≈360 fA, respectively. In Figure 5 C and D the reverse voltages were 50 mV and 300 mV resulting to generated dark currents of ≈100 fA and ≈200 fA for the single photodiode case in C. The results of Figure 5 C confirm the ability of the amperometric channel to decipher current differences of 100 fA upon a baseline of 100 fA. The standard deviation value of the photodiode current in Figure 5 C lies in the range of 60 to 70 fA.

Supporting Table 1 provides an evaluation of the amperometric performance of our ASIC when compared against other recent works focusing on amperometry. Our ASIC compares favourably.

#### 2.2.3 Test Current Input Source–Biosensor

The third and final testing scenario evaluated the performance of the proposed circuit under a more realistic setup with the use of the lactate sensor described in detail in.^32^

The sensor was connected to the low volume, low flow rate, microdialysis stream using a microfluidic PDMS chip. The microfluidic chip was fabricated using soft lithography.^33^

Programmable LabSmith microfluidic pumps and valves (see Figure 3) were used to provide a constant flow of known calibration standards, in a precise order, to the microfluidic chip containing the lactate sensor. Knowing the measured concentration accomplishes two purposes; first allows for the calibration of the sensor by correlating the concentrations to actual current measured values and the second facilitates the presentation of the high performance of the proposed circuit capable to read low differences in concentration.

**Figure 6:**
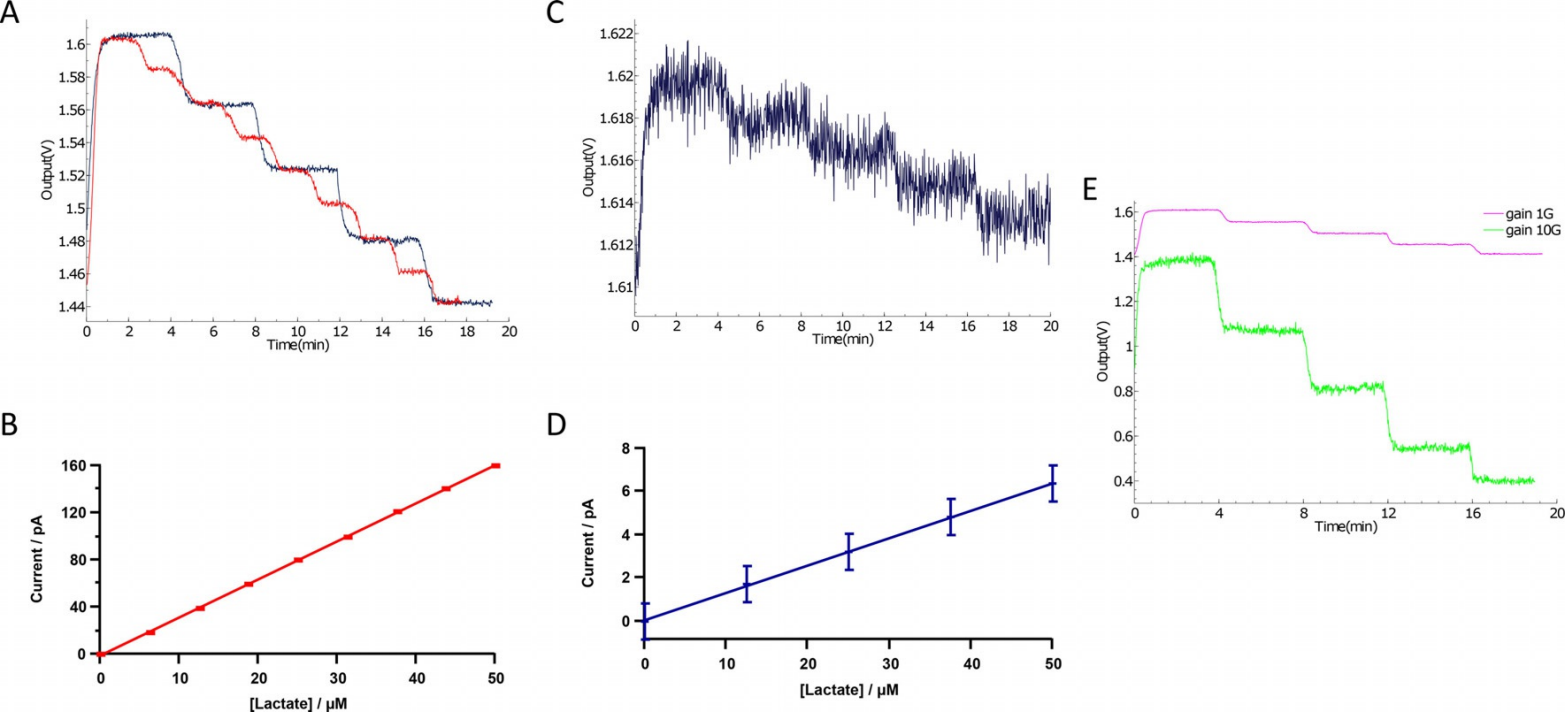
A) Lactate biosensor results using the LabSmith set‐up to vary the lactate concentration starting with 0 μm and increasing the concentration with a step of 12.5 μm (blue) and 6.25 μm (red) until reaching 50 μm lactate. The ASIC measures a voltage change from 1.60 V to 1.44 V, a change of 160 mV, with a 6.25 μm concentration change corresponding to 20 mV at the output. As the transimpedance gain is set to 1 mV pA^−1^ this give the current calibration curve shown in (B). B) Conventional current calibration curnve using data from (A). C) Lactate biosensor from (A) calibrated from 0 μm to 50 μm in 12.5 μm concentration reduction steps. The biosensor shows typical failure through extensive continuous use resulting in decreased sensitivity (transimpedance gain 1 mV pA^−1^ for comparison with A). Note that the changes can still be resolved without additional filtering. D) Conventional current calibration curve using data from C. E) The effect of increasing the transimpedance gain from 1 mV pA^−1^ to 10 mV pA^−1^ for a second lactate biosensor.

For the results presented in Figure 6 A, B the pumps are programmed to vary the lactate concentration in phosphatebuffered saline (PBS) from 0 μm to 50 μm lactate with a step of either 12.5 μm every 4 minutes (blue line) or a step change of 6.25 μm every 2 minutes (red line). The transimpedance gain for both traces is 1 mV pA^−1^. Although the concentration is increasing, the voltage output is decreasing. This behaviour is related to the direction of the measured current. The start voltage of 1.65 V (=half the 3.3 V power supply level of the ASIC) is the output potential equivalent to zero current input to the ASICs amperometry channel. The lactate biosensor produces H_2_O_2_ which is oxidised at working electrode potential of +0.7 V vs. Ag|AgCl reference electrode. The flow of electrons into the ASIC working electrode terminal is equivalent to a nominal current flow (i.e. a flow of positive charges) out of the ASIC. This in turn makes the output voltage of the sensor go down. In general, when electrons enter the ASIC, they are converted to output voltages starting from 1.65 V and falling towards 0 V as the current increases. Conversely, when electrons leave the ASIC terminal, they are converted to output voltages which start from 1.65 V and increase towards the 3.3 V supply level as the current increases. The same biosensor used for the experiment of Figure 6 A, B is calibrated much later after it has lost sensitivity to give 4 C,D. The low noise allows the much smaller current changes from the biosensor to still be resolved without the need for filtering.

Since the measured input lies within the range of −165 pA to 165 pA a gain of 10 mV pA^−1^ can be introduced to amplify more the input current signal. Amplifying the input current more, improves the clarity of the measurement and provides headroom for smaller changes in concentration to be detected. The green line in Figure 6 E represents the response of the circuit for the same variation of concentrations (steps of 6.25 μm) but now with a higher gain value set equal to 10 mV pA^−1^. A reasonable behaviour is observed for concentrations of 17.5 μm and 25 μm. The standard deviation values of the estimated biosensor current values for both gain values lie in range of 1.2 to 1.5 pA. The corresponding limit of detection values lie in the range of 2.5 to 9.5 nm of lactate. This compares to values of 0.4 μm obtained with similar sensors and conventional instrumentation (data not shown). We have on one occasion obtained a detection limit of 10 nm for ATP.^32^ However, this used enzyme amplification to enhance the level of a chemical intermediary, followed by assay. This is, in some ways, a chemical equivalent of the charge storage and amplification mechanism exploited by the SC technique.

Although the initial sensitivity of such biosensors is adequate, the decline after long periods of operation results into performance degradation when the sensed current differences Δ*I* become so small—for the same concentration difference Δ*C*—that cannot be detected reliably. This limitation is often referred to as “sensor drift”, and in practice means that the same concentration value is measured as lower current value after long periods of monitoring compared to the measured value when monitoring started, Figure 6 C. Currently, to counterbalance for the drop in sensitivity manual adjustment of the *I*‐to‐*V* transimpedence gain is usually applied. Here, we address this problem, by using an amperometric channel module which automatically adjusts its transimpedance gain and is tailored for online glucose and lactate monitoring of TBI patients. The automatic transimpedance gain control circuit is suitable for switched‐capacitor based current analogue front‐ends, occupies an area of 0.028 square mm, consumes 14 μW from a 3.3. V power supply and offers automatic allocation between three transimpedance gain values, namely 1, 10 and 100 mV pA^−1^, each one optimised for current sensor value ranges of ±1.65 nA, ±165 pA and ±16.5 pA respectively.

The automatic transimpedence gain control circuit suitable for switched‐capacitor‐based current analogue front ends was designed and tested successfully and can be found in.^34^ This lower current detection ability ensures the gain range is adequate enough so that reliable use of biosensors is prolonged, even as sensitivity fails, thus increasing their longevity in a clinical setting.

### 2.3 Noise Performance of the ASIC

Noise ultimately determines the detection limits. The CDS technique reduces the impact of the flicker noise by moving the corner frequency at lower range. The corner frequency for the amperometric channels of the LENBIC was pushed down to a very low frequency of approximately 200 mHz. The noise floor in the bandwidth of interest (<10 Hz) ranges from 20 fA/√Hz to 50 fA/√Hz. The two ECoG channels have different gains and bandwidths and the flicker noise for both channels is minimised thanks to the applied CDS technique. The corner frequency is 500 mHz. The noise floor at the passband for the ECoG channel A is 50 nV/√Hz and for the ECoG channel B is 80 nV/√Hz. The gain of the K+ channels is optimised to measure signals of greater amplitude compared to the ECoG channel case, hence the noise floor at the passband is 350 nV/√Hz.

### 2.4 Power Consumption of the ASIC

Power consumption is important for a wearable analytical device. We traded‐off power consumption for a better noise performance. The power consumption of the LENBIC could be separated into three major categories. The analogue part, the digital part and the Analog‐to‐Digital Converter (ADC). The analogue part constitutes the major contributor due to its size and the number of channels that are reported (8 ECoG channels, 2 potentiometric channels and 3 amperometric channels). Thus a power consumption of 3.8 mW for the analogue part, considering the amount of channels, is reasonable. The power consumption of the digital part is 1.55 nW and that of the ADC 100 μW. The power consumption of the utilised buffers to connect in‐between stages or to drive the testing points and the biasing circuits, constitutes more than half of the 9.678 mW total power consumption.The detailed power breakdown can be found in supporting Figure 6.

## 3 Conclusions

We present the design, fabrication and testing of the first custom‐made application specific integrated chip (ASIC) design for TBI monitoring. The microchip compromises three current channels for biosensor measurements of glucose and lactate and ten voltage measurement channels for potassium and ECoG. Concurrent changes in these neural and electrical signals have been associated with SD waves, which in turn are strongly associated with a poor patient outcome. The detection of SD waves can lead to the earlier detection (and ultimately may lead its prevention) of secondary brain injury. In this work, we have started with the specifications of the signals of interest (neurochemical and electrophysiological) and used these to define the specifications and the target performance of the chip. We have included real life situations, such as the inevitable fall in sensitivity of real world biosensors. The basic architecture is modular, allowing for easy generation of new ASICs targeting different physiological processes.

**Figure 7:**
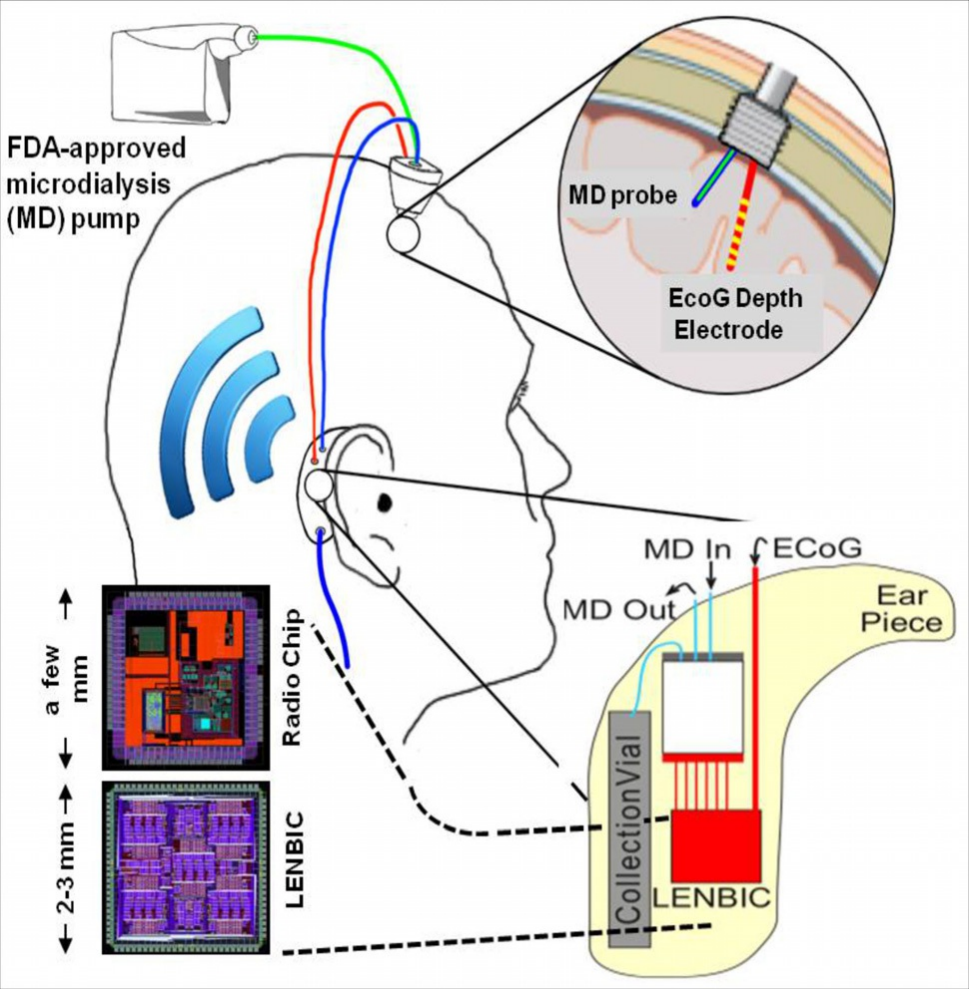
Proposed “behind‐the‐ear” microplatform for TBI monitoring.

In future, the LENBIC ASIC presented here will be used as the core processor of a “behind the ear” wireless microplatform, which could enable the monitoring of a greater portion of patients suffering from TBI. Such patient populations include a) those who have undergone craniotomy neurosurgery are awaken from sedation in the ICU and become mobilised, and b) those who were judged not in need of surgery but are admitted in high dependency units remaining mobile. Both these categories of patients are currently not monitored for SD waves. More specifically, as illustrated in Figure 7 the “behind‐the‐ear” microplatform will comprise(/support) the following components(/actions):

- An appropriately constructed/modified cranial bolt through which a microdialysis probe and a depth spencer (ECoG) electrode will be monitoring the targeted chemical and electrical physiological parameters of the human brain tissue.
- A miniaturised integrated analysis microfluidic chip that can fit into a patient‐mounted “behind‐the‐ear” module together with the microelectronic chips.
- Electrical and fluidic connections from the integrated analysis chip are input to our Low‐power Electrical and Neurochemical Biosensor Interfacing (microelectronic) Chip (LENBIC)
- A radio transceiver (microelectronic) chip which transmits all the digitally‐converted TBI data to a nearby station (or to cloud) for further post‐processing. The miniaturised radio transceiver should operate in one of the license‐free ISM radio bands (e.g., at 2.45 GHz) and the adopted radio protocol could be one suitable for short‐range (e.g., able to cover a hospital ward) and particularly robust to other sources of radio interference (e.g., zigbee protocol). Ceramic antenna can be used to preserve the low area profile of the microplatform.

The above stress the key role of the TBI ASIC presented in this work as the core moduel for the realisation of the envisioned wireless TBI micro‐modality we are working on. With this miniaturised platform, a cranial bolt can be used on a wider patient set, which could include mobile patients. It is believed that such a modality has the potential to alter the TBI patient's pathway enabling their monitoring irrespectively of whether they are in a sedated or mobile state and will facilitate the collection of valuable clinical data.

## Experimental Methods

### The Low‐Power Electrical and Neurochemical Biosensor Interfacing Chip

The Low‐power Electrical and Neurochemical Biosensor Interfacing Chip (LENBIC) is an application specific integrated circuit (ASIC) designed and optimised to monitor TBI related signals.

**Figure 8:**
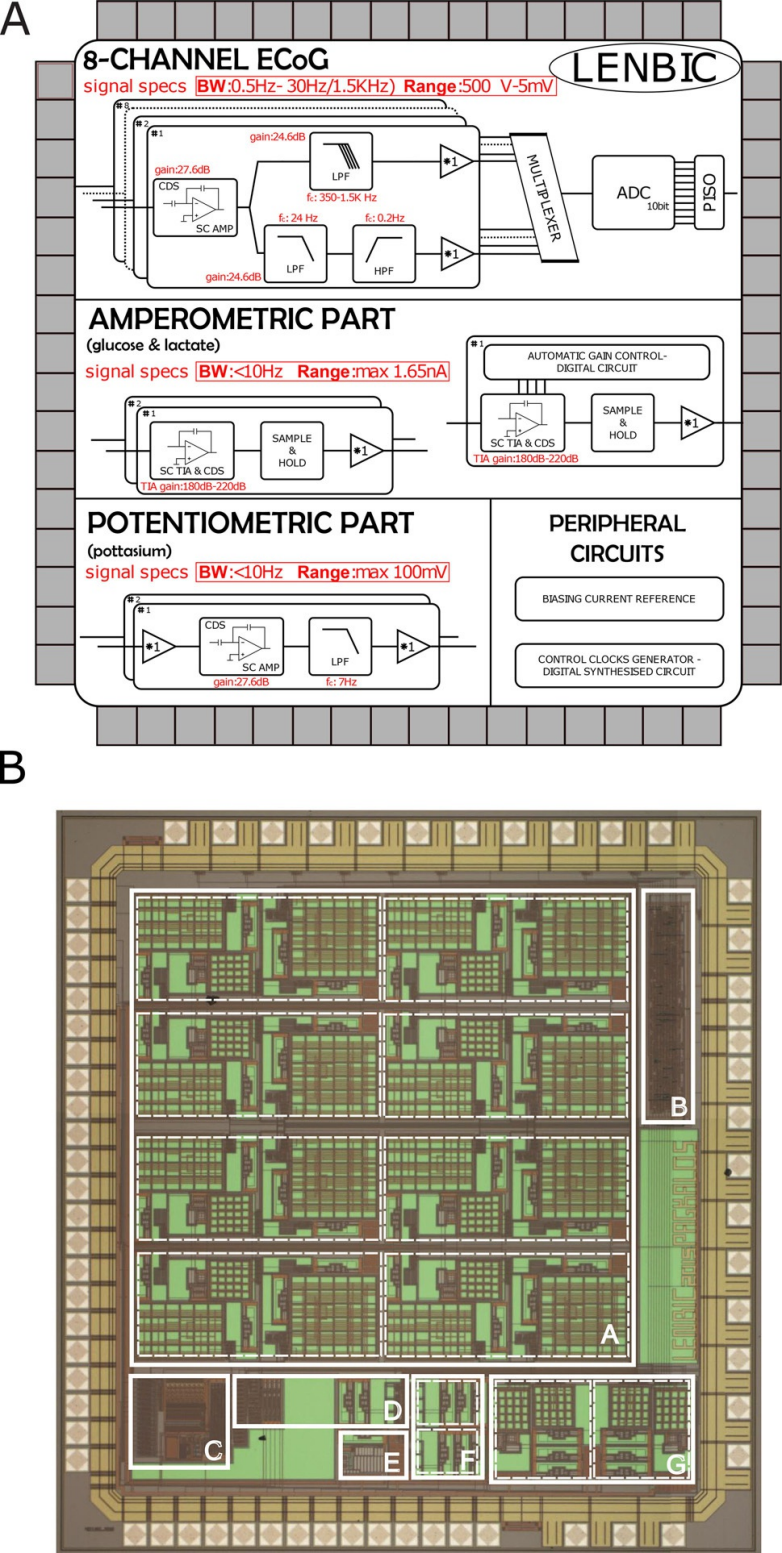
A) Microchip block diagram of LENBIC parts. B) Microphotograph of the fabricated LENBIC microchip occupying a total area of 7.5 mm^2^. The white squares indicate the area of the specific parts: A is the array of the eight ECoG processing channels, B is the digital circuit which generates the control clocks, C is the multiplexer (MUX) and ADC interface which digitises the ECoG channels, D is the automatic gain control module, E is the biasing circuit, F are the two amperometric channels and G are the two potassium processing channels.

The information simultaneously generated by the sensors measuring the chemicals and the electrodes measuring the ECoG signal, call for different signal processing methods. Chemicals, such as glucose and lactate, are measured by means of amperometric biosensors and thus translate the measured concentration into current. In contrast potassium and brain activity sensors, generate voltage. More specifically, the LENBIC has been divided into two main parts: the current analogue front end (AFE) and the voltage AFE, which are accompanied by periphery circuits to support their function. Figure 8 illustrates the operational block diagram and a microphotograph of the fabricated LENBIC chip, which includes two amperometric channels to record changes in glucose and lactate levels with one optimised amperometric channel to automatically control its transconductance gain,^34^ eight voltage processing channels for ECoG monitoring, two potentiometry channels to record changes in potassium levels, a current biasing module, decision circuits for digital control, digital circuits to generate control clocks, and a multiplexing ADC to transfer the recorded ECoG signals to a PC interface. This paper will focus on the voltage AFE for ECoG and the current AFE for amperometric measurements as these are the most challenging aspects and the correct operation of potentiometric circuitry for potassium monitoring has been already been confirmed.

### Test Voltage Measurements for ECoG

Much research in recent years has focused on voltage processing AFEs realising miniaturised monitoring systems for monitoring biopotential signals such as EEG, ECoG, ECG and EMG. The proposed voltage AFE is a cascaded design, composed of an amplifier and filters and as first proven in,^35^ the noise Figure of a cascaded topology is heavily depended on the noise Figure of the first stage.

In this work a low noise Switched‐Capacitor (SC)^36^ amplifier was employed as the first stage of the voltage AFE: supporting Figure 2 illustrates the topology of the corresponding amplifier used in both ECoG and K channels along with the corresponding list of specifications of the block and its transfer function. A single stage Operational‐Transconductance Amplifier (OTA) was preferred instead of a two stage implementation, targeting lower levels of power consumption and better noise performance. The linearity of the first stage is not critical since the amplification introduced at this stage satisfies the requirements for TBI. Flicker noise dominates at low frequencies, however the Correlated Double Sampling (CDS) technique^37^ has been utilised in order to minimise the flicker noise and errors due to finite offset voltages and finite opamp gain. For this reason it was preferred to target lower thermal noise by using an NMOS differential pair, while its increased flicker noise was neglected by CDS.

SC filters are another application of the SC circuits.^38^ The main advantage of this implementation, compared to other types of filters, is that with SC circuits the gain and the cut‐off frequency of the filter are precisely determined by monolithically realised capacitor ratios (ratiometric design). In addition, the capacitor ratio value can be easily tuned by changing the capacitor size thus tuning the gain and the cut‐off frequency. In this work a first order SC low pass filter was used as a second stage for the voltage processing channels.

The proposed SC low pass filter, shown in supporting Figure 3, has been used for both the ECoG and potassium concentration signals and the cut‐off frequencies of the low pass filter were dictated by the signals of interest. In the ECoG signal processing channel, in order to capture efficiently the two frequency bands of interest (the SD waves and higher frequency brain activity) two separate filters were required after the common stage of CDS low noise SC amplifier. These two different filters were termed ECoG Ch A and ECoG Ch B, respectively. Supporting Figure 3 also provides the specifications and the transfer function of the first order SC low pass filter used as a second stage in ECoG ChA, ECoG ChB and in K channel.

Supporting Figure 4 illustrates the measured frequency response of the K channel. It has been confirmed that the buffered cascade (see the potentiometric part of the LENBIC in Figure 8 A) of the amplifier (shown in supporting Figure 2) followed by an appropriately tuned topology of our first order SC low pass filter (supporting Figure 3) lead to a low frequency gain of 27dB (bear in mind specifications in supporting Figure 2) and a−3dB bandwidth of 7 Hz. It should be stressed that the potentiometric channels are preceded by on‐chip buffers (i.e. we have built on the chip high input‐impedance voltage followers) which ensure the appropriate interfacing with high output impedance potentiometric sensors.

### Test Current Measurements for Amperometry

Continuous and discrete time design approaches both offer high performance implementations but with certain limitations^39^ and the choice regarding which approach to follow relies on the specifications of the targeted application. For TBI monitoring, a discrete time approach was adopted and optimised to achieve high performance measurements of the low frequency target signals.

SC circuits are widely used in filters, comparators, DACs and ADCs.^36^ The scope of the proposed amperometric part of the microchip was to realise a SC circuit capable of detecting low current inputs with an optimised performance (supporting Figure 5). Compared to the conventional feedback resistor‐based TransImpedance Amplifier (TIA), in the case of SC TIA the resistor in the feedback loop is replaced by a capacitor in parallel with a switch. By controlling the capacitor reset time (which is essential in order to prevent the integrator from saturation) and the value of the capacitor, their ratio can be viewed as the impedance of the feedback loop. By controlling this ratio effectively, a variety of input current ranges can be detected. Supporting Figure 5 details the transimpedance offered by the amperometric AFE topology shown, along with temporal phase information and chosen capacitor values. In this implementation the value of the capacitor was set at 1 pF and the integration time was set initially at 1 ms, leading to an effective resistance of 1GΩ.

The second stage of the proposed current AFE, consists of the CDS feature and the Sampling‐and‐Hold (S/H) stage, both of which have a significant role in processing the voltage output from the first stage. The CDS is a well known technique that is being used in SC circuits to minimize errors due to finite offset voltages, flicker noise (1/*f*) and finite OPAMP gain^37^, ^40^ and it has been used in SC gain amplifiers, integrators and S/H. The basic methodology is similar in all cases : during a calibration phase, the finite input voltage of the opamp is sampled and stored across capacitors; during the operation phase (when the output is being sampled and held), this error voltage is subtracted from the signal voltage by appropriate switching of the capacitors. The CDS technique was chosen among other techniques, such as auto‐zeroing and chopper stabilisation technique, which also minimise the noise levels.^37^, ^41^, ^42^ The main reason for this choice was that the noise reduction effect of these techniques in correlation with the increase in power consumption, was considered inefficient for the low frequency range of interest of our particular TBI application. Thus the CDS technique was adopted.

### Peripheral Circuitry

The peripheral circuitry supports the operation of incorporated AFEs on LENBIC and includes: a biasing reference current module to generate the biasing currents of the utilised opamps; a digitally synthesised module to generate the control clocks for the SC circuits; and finally an interface to convert the analogue value of the 16 channels into a digital form. To achieve this, a system combining a multiplexer (MUX), an ADC and a Parallel Input Serial Output (PISO) shift register, was implemented to process the information in the desired form. The ADC is a typical 10‐bit Successive Approximation Register (SAR) type provided by standard circuit libraries of Austria MicroSystems (AMS), the commercially available CMOS technology used for fabricating LENBIC.^43^ The design and development of an optimised ADC was not in the scope of this work.

For reasons of completeness we provide additional, detailed transistor‐level information on the LENBIC: Supporting Figure 6 illustrates the schematics and transistor sizes of the opamp used for the realisation of the amperometric AFE (shown in supporting Figure 5) and of typical switches used in the control of the temporal phases of the amperometric AFE. The same supporting Figure lists the area‐ and power‐breakdown of the different circuit parts of LENBIC. The information provided in supporting Figures 2–6 aims, among others, at facilitating the reproduction of the LENBIC design by interested researchers/parties. Full details can be found in Ref. 44.

### Biosensor Construction

Teflon insulated 50 μm platinum wire (AM Systems Inc) and polyester insulated 50 μm silver wire (AM Systems Inc) were threaded through a hypodermic needle. The platinum disc was used as the working electrode and the silver disc was chloridised using a referencing solution (BASi Inc) to create a Ag|AgCl reference electrode. The needle shaft acted as a counter electrode. The needle was sealed with an epoxy resin (Robnor Resin) and the electrodes were polished using varying grades of alumina slurries. All the chemicals are obtained from Sigma–Aldrich (UK) unless otherwise stated. To fabricate the biosensors for glucose and lactate, first an interference layer, *m*‐phenylenediamine (mPD) film (100 mm in phosphate buffered saline, pH 7.4), was electropolymerised onto the working electrode surface. A second layer was added for specificity to glucose and lactate, and consists of a polyethylene glycerol hydrogel loaded with enzyme (substrate oxidase) that is dip coated onto the needle electrodes.^45^ The biosensor was operated at +0.7 V vs. Ag|AgCl reference.

### Microfluidic Chip Construction

PDMS elastomer (Sylgard 184) was thoroughly mixed in a 10:1 ratio and poured over an SU8 master. The mixture is degassed and cured for 1 hour at 65 °C. The PDMS was peeled away from the master, cut into individual chips and access holes were created using a biopsy punch. The chips were placed on a semi‐cured base of PDMS and baked overnight at 65 °C to ensure bonding of layers.

## Conflict of interest

*The authors declare no conflict of interest*.

## Supporting information

As a service to our authors and readers, this journal provides supporting information supplied by the authors. Such materials are peer reviewed and may be re‐organized for online delivery, but are not copy‐edited or typeset. Technical support issues arising from supporting information (other than missing files) should be addressed to the authors.

Supplementary
