# Supplementary material for: A High‐Performance Application Specific Integrated Circuit for Electrical and Neurochemical Traumatic Brain Injury Monitoring
Source: Chemphyschem. 2018 Mar 9;19(10):1215–25. doi: 10.1002/cphc.201701119 (PMC6016079; doi:10.1002/cphc.201701119)
Supplement: Supplementary file 1 — Supplementary [file CPHC-19-1215-s001.pdf]

CHEMPHYSCHEM

Supporting Information

A High-Performance Application Specific Integrated Circuit for Electrical and Neurochemical Traumatic Brain Injury Monitoring

Ilias Pagkalos, Michelle L. Rogers, Martyn G. Boutelle,* and Emmanuel M. Drakakis*[a]

cphc_201701119_sm_miscellaneous_information.pdf

Author Contributions

Ilias Pagkalos designed, simulated, laid-out, verified and tested the TBI chip electrically and interfaced with biosensors; wrote and edited the manuscript.
Michelle L. Rogers constructed the biosensors, interfaced the biosensors with the TBI chip and supported testing of chip with biosensors; wrote and edited the manuscript.
Martyn G. Boutelle guided biosensor construction and testing; wrote and edited the manuscript.
Emmanuel M. Drakakis conceived the TBI chip and guided its design, realisation and testing; wrote and edited the manuscript.

Supplementary figures

Supplementary Figure 1

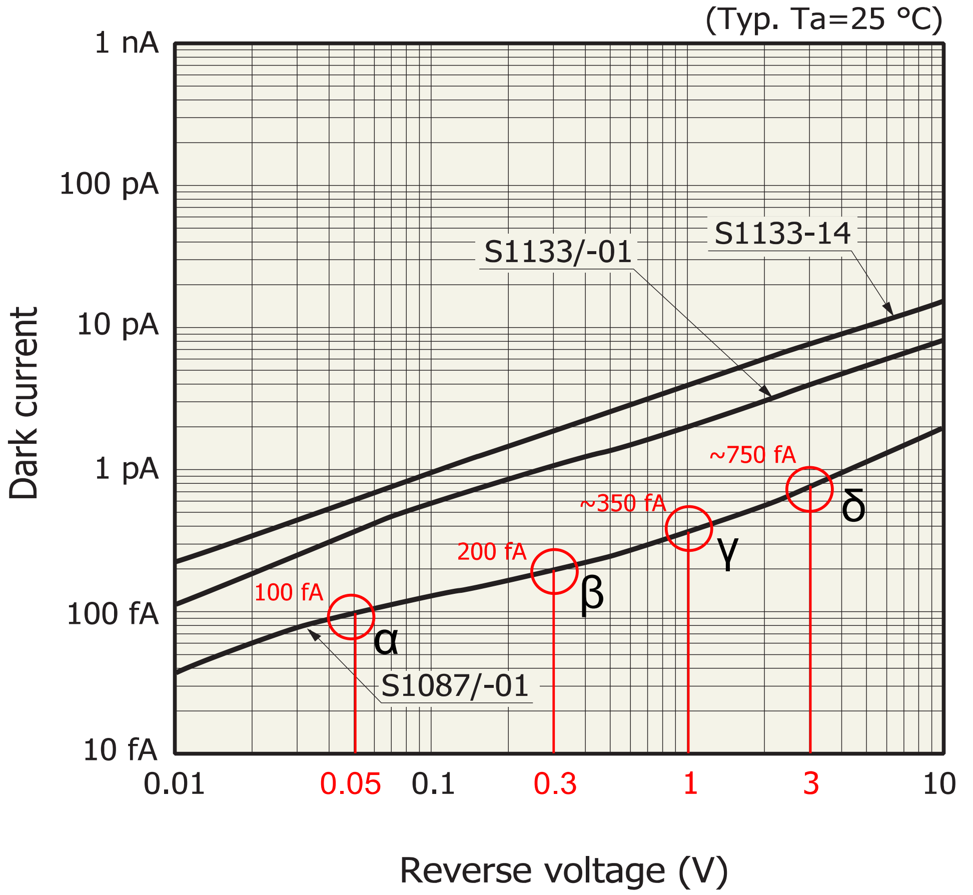

Dark current characteristic of the Hamamatsu S1087 photodiode; extracted from [29]

Supplementary Figure 2

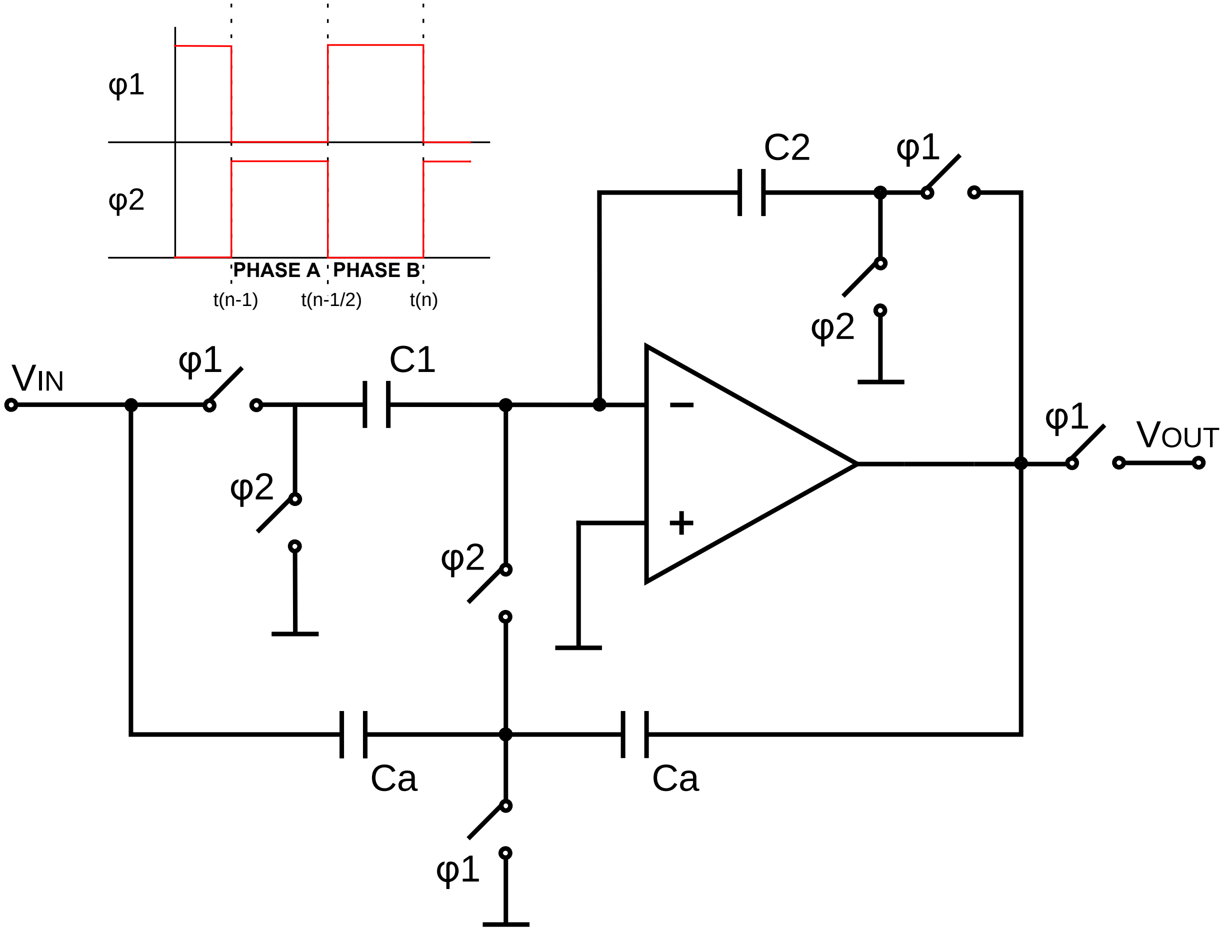

Transfer-Function

$$\frac{V_{OUT}(n)}{V_{IN}(n)} = -\frac{C_1}{C_2}$$

Specifications

	ECoG Ch & K+ Ch
f_s	$100KHz$
C_1	$12pF$
C_2	$500fA$
C_a	$500fA$
$gain$	$27.6dB$

Low-noise SC amplifier used as first stage in both ECoG channels and K+ channel; timing diagram, transfer function and specifications.

Supplementary Figure 3

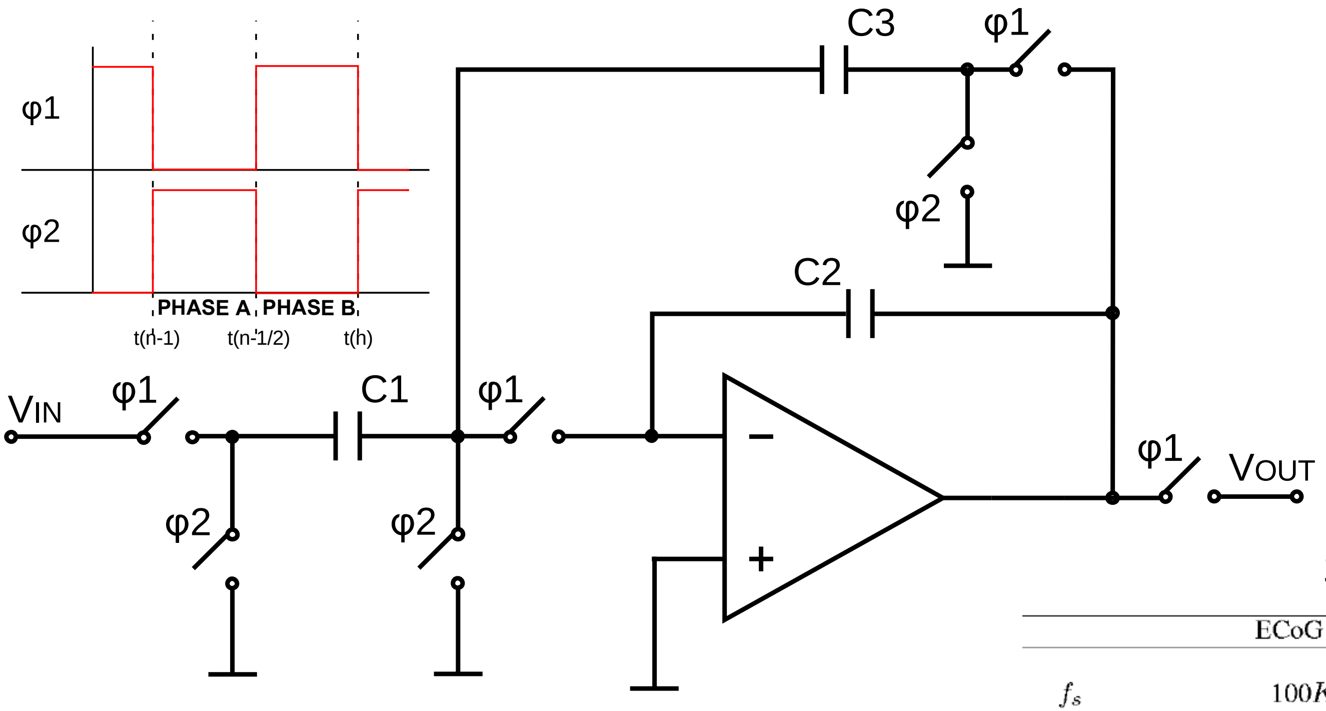

Transfer-Function

$$\frac{V_{OUT}(j\omega)}{V_{IN}(j\omega)} = \frac{C_1}{C_3}\frac{1}{1+j\omega\frac{TC_2}{C_3}} \Leftrightarrow \frac{V_{OUT}(j\omega)}{V_{IN}(j\omega)} = \frac{C_1}{C_3}\frac{1}{1+j\omega\frac{TC_2}{C_3}}$$

Specifications

	ECoG Ch A	ECoG Ch B	K+ Ch
f_s	$100KHz$	$10KHz$	$1KHz$
C_1	$6pF$	$8pF$	$500fF$
C_2	$16pF/8pF/5.33pF/3.2pF$	$24pF$	$500fF$
C_3	$350fF$	$350fF$	$500fF$
f_c	$350Hz/750Hz/1050Hz/1550Hz$	$24Hz$	$7Hz$
$gain$	$24.7dB$	$27.2dB$	$0dB$

Topology of utilised first-order SC low pass filter used as second stage in ECoG Ch A, ECoG Ch B and in K+ Ch; timing diagram, transfer function and specifications.

Supplementary Figure 4

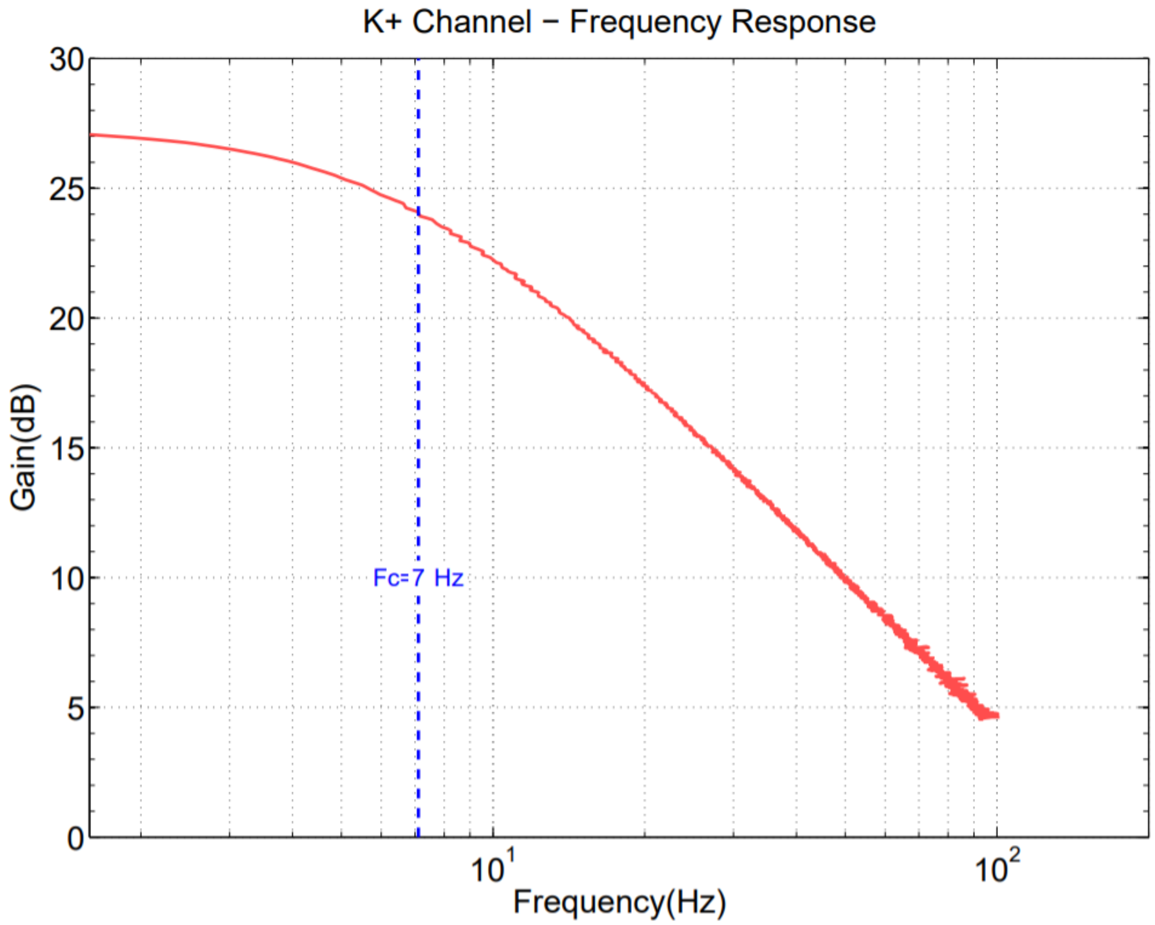

Typical measured frequency response of the K+ channel characterised by a low frequency gain of 27dB and a -3dB bandwidth of 7Hz.

Supplementary Figure 5

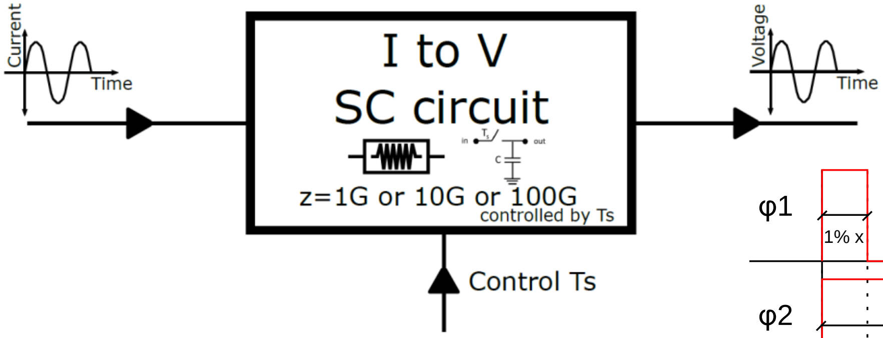

Operation

- In phase A ($\phi 1$, $\phi 2$ close; $\phi 3$, $\phi 4$ open) C_F is in reset mode while C_1 captures any noise introduced by the switching of $\phi 1$.
- In phase B ($\phi 2$ closes; $\phi 1$, $\phi 3$, $\phi 4$ open) the integration of the input starts and C_1 is charged with the noise level at the start of this integration.
- In phase C ($\phi 1$, $\phi 2$, $\phi 3$, $\phi 4$ open) the integration continues.
- In phase D ($\phi 4$ close; $\phi 1$, $\phi 2$, $\phi 3$ open) C_H is in reset mode.
- In phase E ($\phi 3$ closes; $\phi 1$, $\phi 2$, $\phi 4$ open) the charge after one period of integration will be cleared of noise introduced in the beginning, amplified and sampled and held on C_H.

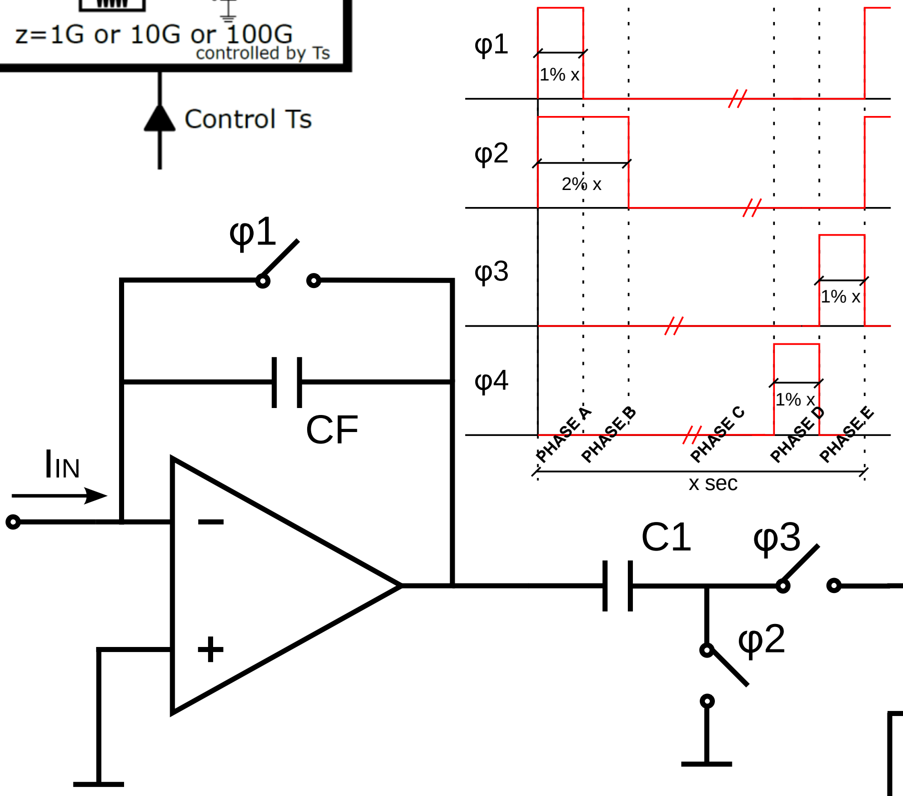

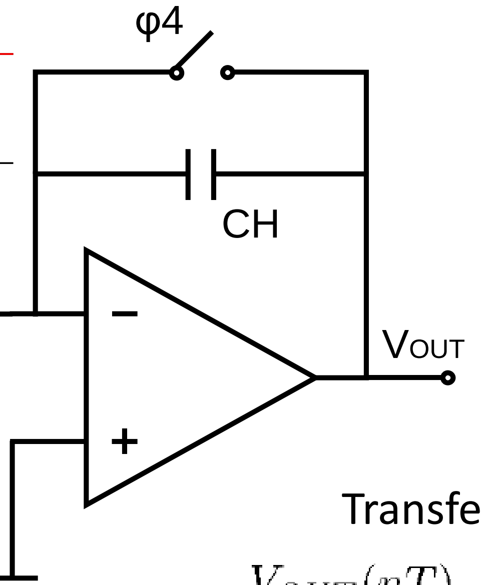

Proposed current AFE, incorporating a SC TIA stage, a CDS stage and a S/H stage. The block diagram on the top left is a simplistic representation of the current AFE and its operation, converting current into voltage with a variable impedance 1G or 10G or 10G Ohm set by controlling the timing TS of the switches. The timing inset graph presents the switches' control signals of the proposed SC circuit, where x is the time of one period varying as 1ms, 10ms and 100ms according to the desired transconductance gain of 1G, 10G and 100G, respectively.

Transfer-Function

$$\frac{V_{OUT}(nT)}{I_{IN}(nT)} = \frac{T_{INT}\, C_1}{C_F\, C_H}$$

T_{INT} :integration time set to 1,10,100 msec (x in phase diagram)

C_1, C_F, C_H : 1pF

Supplementary Figure 6

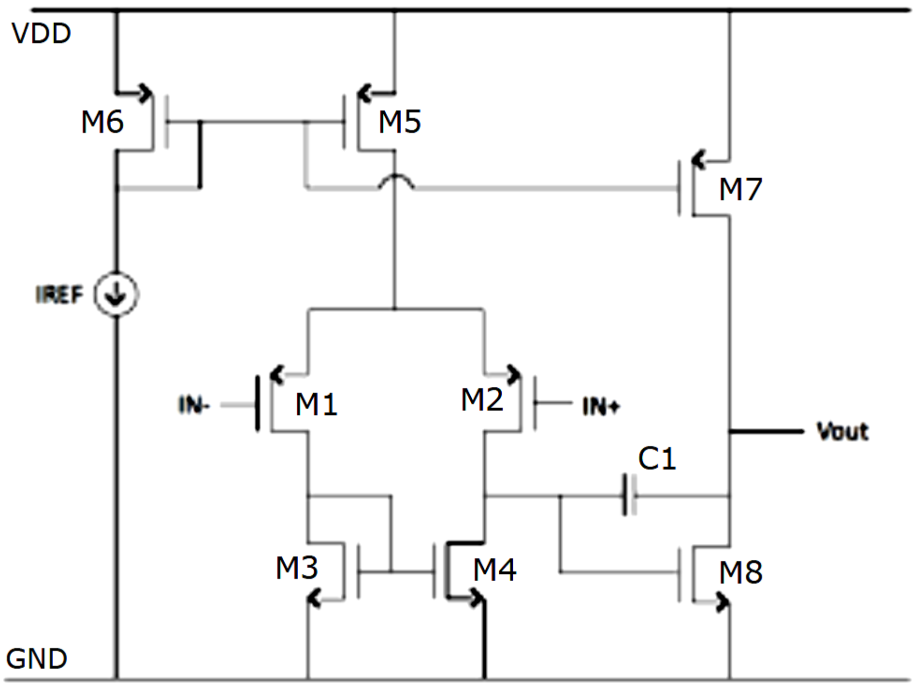

Two stage Miller OPAMP topology of the utilized SC circuits of this work. Summarized specifications:

IREF	10nA
C1	3pF
VDD	3.3V

Transistor	W[um]/L[um]
M1,M2	50/1
M3,M4,M5,M6,M7,M8	20/1

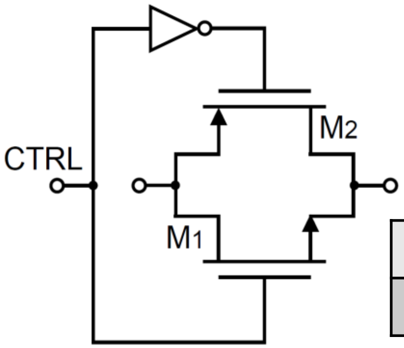

Transmission Gate switch of the utilized SC circuits of this work

Transistor	W[um]/L[um]
M1,M2	1/0.35

Block	Power Consumption
CDS SC Amplifier	36 μW
SC LPF - ECoG ChA	75 μW
SC LPF - ECoG ChB	75 μW
SC LPF - K+ Ch	75 μW
Current AFE	180 μW
ADC	100 μW
Digital Synthesised Clocks	1.55nW
Biasing/Buffering/Testing structures	5.831 mW
Total	9.678 mW

Block	Area $[mm^2]$
CDS SC Amplifier	0.0576
SC LPF - ECoG ChA	0.1610
SC LPF - ECoG ChB	0.1104
SC LPF - K+ Ch	0.0598
HPF	0.0123
Current AFE	0.0500
Automatic Gain Control Module	0.0260
PISO	0.0135
MUX	0.0300
ADC	0.0832
Digital Clocks Generator	0.1570
Total	7.5 mm^2

Power consumption and area breakdown of the LENBIC blocks. LENBIC has been fabricated in the AMS 0.35um CMOS process, occupies an area of 7.5mm2 and is powered by 3.3V.

Supplementary Table 1

Comparison Table of Current AFEs

Author-Paper Year	Heitz [1] 2011	Wang [2] 2010	Bennati [3] 2009	Ayers [4] 2007	Ferrari [5] 2009	Sampietro [6] 2009	**This work** 2017
Technology	$0.18\mu m$	$0.35\mu m$	$0.35\mu m$	$0.5\mu m$	$0.35\mu m$	$0.35\mu m$	$0.35\mu m$
Supply Voltage	-	-	-	-	-	-	$3.3\ V$
Bandwidth	$800Hz$	$5KHz$	$4KHz$	$2KHz$	$4MHz$	$1MHz$	$10Hz$
Noise Floor	$150fA/\sqrt{Hz}$	$7fA/\sqrt{Hz}$	$5fA/\sqrt{Hz}$	$3fA/\sqrt{Hz}$	$3fA/\sqrt{Hz}$	$0.5fA/\sqrt{Hz}$	$20fA/\sqrt{Hz}$
Power Consumption	$2.4mW$	-	$15mW$	$1\mu W$	$60mW$	$55mW$	$180\mu W$
Approach	DT	DT	DT	DT	CT	CT	DT
Transient Amperometry Profiles	✓	✓	✓	✓	✗	✗	✓
Transient Concentration Profiles	✓	✓	✓	✗	✗	✗	✓
*M*inimum recorded current shown in graph	$5pA$	$3pA$	$60pA$	$10pA$	-	-	$100fA$
Glucose/Lactate detection	✗	✗	✗	✗	✗	✗	✓
Ampero&Potentio on the same chip	✗	✗	✗	✗	✗	✗	✓

[1] R. T. Heitz, D. B. Barkin, T. D. O'Sullivan, N. Parashurama, S. S. Gambhir, B. A. Wooley, A low noise current readout architecture for fluorescence detection in living subjects, Solid-State Circuits (2011) 308–310.

[2] G. Wang, W. B. Dunbar, An integrated, low noise patch-clamp amplifier for biological nanopore applications, in: Engineering in Medicine and Biology Society (EMBC), 2010 Annual International Conference of the IEEE, 2010, pp. 2718–2721.

[3] M. Bennati, F. Thei, M. Rossi, M. Crescentini, G. D'Avino, A. Baschirotto, M. Tartagni, 20.5 A Sub-pA ΔΣ Current Amplifier for Single-Molecule Nanosensors, in: Solid-State Circuits Conference - Digest of Technical Papers, 2009. ISSCC 2009. IEEE International, 2009, pp. 348–349.

[4] S. Ayers, K. D. Gillis, M. Lindau, B. A. Minch, Design of a CMOS Potentiostat Circuit for Electrochemical Detector Arrays, Circuits and Systems I: Regular Papers, IEEE Transactions on 54 (4) (2007) 736–744.

[5] G. Ferrari, F. Gozzini, A. Molari, M. Sampietro, Transimpedance Amplifier for High Sensitivity Current Measurements on Nanodevices, IEEE Journal of Solid-State Circuits 44 (5) (2009) 1609–1616.

[6] G. Ferrari, M. Farina, F. Guagliardo, M. Carminati, M. Sampietro, Ultra-low-noise CMOS current preamplifier from DC to 1MHz, Electronics Letters 45 (25) (2009) 1278.

*DT : Discrete Time

**CT : Continuous Time
